# Innovations in gender affirmation: AI-enhanced surgical guides for mandibular facial feminization surgery

**DOI:** 10.1007/s00784-025-06459-2

**Published:** 2025-07-25

**Authors:** Michel Beyer, Sead Abazi, Céline Tourbier, Alexandru Burde, Shankeeth Vinayahalingam, Robert R. Ileșan, Florian M. Thieringer

**Affiliations:** 1 Department of Oral and Cranio-Maxillofacial Surgery and 3D Print Lab, University Hospital Basel, Basel, Switzerland; 2 Medical Additive Manufacturing Research Group (Swiss MAM), Department of Biomedical Engineering, University of Basel, Allschwil, Switzerland; 3 Department of Prosthetic Dentistry and Dental Materials, Iuliu Hatieganu University of Medicine and Pharmacy, 32 Clinicilor Street, Cluj-Napoca, 400006 Romania; 4 Department of Oral and Maxillofacial Surgery, Radboud University Medical Center, Nijmegen, the Netherlands; 5 Department of Oral and Maxillofacial Surgery, Lucerne Cantonal Hospital, Spitalstrasse, Lucerne, 6000 Switzerland

**Keywords:** Digital workflow, Facial feminization surgery, Cutting guide, Artificial intelligence, Cranio-maxillofacial surgery, Personalized medicine

## Abstract

**Objectives:**

This study presents a fully automated digital workflow using artificial intelligence (AI) to create patient-specific cutting guides for mandible-angle osteotomies in facial feminization surgery (FFS). The goal is to achieve predictable, accurate, and safe results with minimal user input, addressing the time and effort required for conventional guide creation.

**Materials and methods:**

Three-dimensional CT images of 30 male patients were used to develop and validate a workflow that automates two key processes: (1) segmentation of the mandible using a convolutional neural network (3D U-Net architecture) and (2) virtual design of osteotomy-specific cutting guides. Segmentation accuracy was assessed through comparison with expert manual segmentations using the dice similarity coefficient (DSC) and mean surface distance (MSD). The precision of the cutting guides was evaluated based on osteotomy line accuracy and fit. Workflow efficiency was measured by comparing the time required for automated versus manual planning by expert and novice users.

**Results:**

The AI-based workflow achieved a median DSC of 0.966 and a median MSD of 0.212 mm, demonstrating high accuracy. The median planning time was reduced to 1 min and 38 s with the automated system, compared to 19 min and 37 s for an expert and 26 min and 39 s for a novice, representing 10- and 16-fold time reductions, respectively.

**Conclusions:**

The AI-based workflow is accurate, efficient, and cost-effective, significantly reducing planning time while maintaining clinical precision.

**Clinical relevance:**

This workflow improves surgical outcomes with precise and reliable cutting guides, enhancing efficiency and accessibility for clinicians, including those with limited experience in designing cutting guides.

**Supplementary Information:**

The online version contains supplementary material available at 10.1007/s00784-025-06459-2.

## Introduction

The transgender demographic, representing 0.02–0.5% of adult populations [1], seeks surgical interventions to align their physical characteristics with their gender identity. In 2019, approximately 1597 transfeminine individuals in Germany underwent gender-affirming surgical procedures [2]. Although gender diversity is a universal and non-pathological characteristic of humanity [1, 3], gender incongruence can lead to gender dysphoria [4], a condition acknowledged within the International Classification of Diseases and Related Health Problems [5]. Individuals with gender dysphoria often experience discomfort with their anatomical features and societal implications, negatively impacting psychological well-being [6, 7]. This distress often requires interventions, such as gender reassignment procedures, to improve quality of life [8].

Facial feminization surgery (FFS) involves procedures to feminize masculine traits, supporting transgender patients in their transition. These procedures are proven to be safe, effective, and enhance quality of life [9, 10]. Research reveals that FFS increases accurate gender recognition in artificial intelligence systems [11] and reduces societal misgendering, contributing to an improved perception of femininity [12]. Post-operative outcomes consistently report improved psychological health and reduced dysphoria [13–16]. A key aspect of FFS is feminizing the mandible by altering its size and shape through osteotomies and ostectomies, often with the help of patient-specific cutting guides [17]. Achieving a symmetrical and aesthetically pleasing contour is particularly challenging when using a transoral surgical approach, as the limited visibility of the operative field complicates the precise execution of osteotomies [18]. Patient-specific cutting guides generated through virtual surgical planning (VSP) facilitate accurate translation of the preoperative plan to the surgical site, ensuring high precision and reproducibility even under restricted visual conditions. The amount of bone removed during mandibular angle reduction is individually determined based on the patient’s anatomy and the clinical experience of the surgical team. While the use of cutting guides requires a slightly increased soft-tissue detachment compared to freehand surgery, the extent of denudation is not substantially higher, and the overall invasiveness of the procedure remains unchanged. Importantly, the intraoral access avoids visible external scars, while the predefined osteotomy path enhances surgical safety by minimizing the risk of inferior alveolar nerve injury [19]. Thus, the combination of VSP and cutting guides addresses the limitations of intraoral access, supports consistent and symmetrical outcomes, and optimizes both functional and aesthetic results in facial feminization surgery [17–19]. The only notable drawback is the additional time required for the virtual planning and fabrication of the guides.Advancements in virtual surgical planning (VSP) have transformed preoperative planning for FFS, improving safety and accuracy over traditional methods [17, 20, 21]. Cutting guides (CG) are proven to be reliable and precise, though their production remains labor-intensive and often outsourced [22, 23].

This study introduces a fully automated digital workflow that integrates two key innovations: (1) automated segmentation of the mandible using a convolutional neural network (CNN), which extracts the mandible from CT images with minimal manual input, and (2) automated design of patient-specific cutting guides based on the segmented mandible. By automating both the segmentation and guide creation processes, the workflow significantly reduces the time and effort required compared to traditional manual methods, while improving the reproducibility and consistency of cutting guide production. This paper highlights the impact of AI-driven automation on FFS workflows, particularly in enhancing the efficiency and accuracy of surgical planning. With this approach, clinicians—including those with limited experience in CAD/CAM software—can independently perform complex preoperative planning, streamlining the process and reducing dependency on external services.

## Materials and methods

A total of 30 head and neck CT scans were selected from the publicly available dataset in “The Cancer Imaging Archive” (TCIA), which contains 627 Digital Imaging and Communications in Medicine (DICOM) files from head and neck squamous cell carcinoma (HNSCC) patients [24–27]. For this study, only CT scans from male patients were included, as the virtual planning is tailored to facial feminization surgery (FFS), which is typically performed on male individuals transitioning to a female phenotype. The bone reduction in the gonial region and the corresponding surgical guide are specifically intended for use in facial feminization procedures. This subset included 20 images without metallic artifacts and 10 with metallic artifacts, to evaluate the robustness of the workflow under various input conditions. The slice thickness varied from 0.5 mm to 1.0 mm (mean 0.98 mm), and the number of slices ranged from 230 to 549, with a mean of 305.

The workflow is divided into four key steps: segmentation, osteotomy line definition, cutting guide design, and 3D printing. Both the segmentation process and the design creation of the cutting guide are automated. This automated workflow is compared to the traditional manual workflow. The primary outcomes measured include workflow success, time to completion, and the fit of the cutting guide, while secondary outcomes focus on the accuracy of AI-based segmentation.

### Automatic workflow

The automatic workflow introduces two key innovations compared to the manual workflow: automated segmentation of the mandible and automated cutting guide design.

#### Mandible segmentation

Mandible segmentation was performed using a convolutional neural network (CNN) based on the 3D U-Net architecture introduced by Isensee et al. [28]. The model architecture and training strategy closely followed our previously published work on mandible segmentation [29], where a parameter optimization had already been performed. In the present study, the model was trained with similar parameters without further hyperparameter tuning or the use of a separate validation dataset. Specifically, 160 head and neck computed tomography (CT) images from a publicly available database (McGill University, Montreal, Canada) [30] were used for training. The 3D U-Net architecture was employed, incorporating a dice cross-entropy loss function, a depth of 5 layers, a learning rate ranging from 0.0001 to 0.00001, and a patch size of 96 × 96 × 96. The images were resampled to an isotropic voxel spacing of 1.0 × 1.0 × 1.0 mm and clipped to exclude Hounsfield values outside the range of 50–3071. The model was trained for 1000 epochs to optimize segmentation performance specifically for head and neck CT imaging.

The trained 3D U-Net was used for the automated mandible segmentation of the 30 selected head and neck CT scans. The resulting segmentation was automatically imported into Mimics, allowing users to review and manually adjust the segmentation in cases where errors occurred. The mask was then converted into an object within Mimics and transferred to 3-Matic for the next step of cutting guide design creation.

#### Cutting guide design creation

**Fig. 1:**
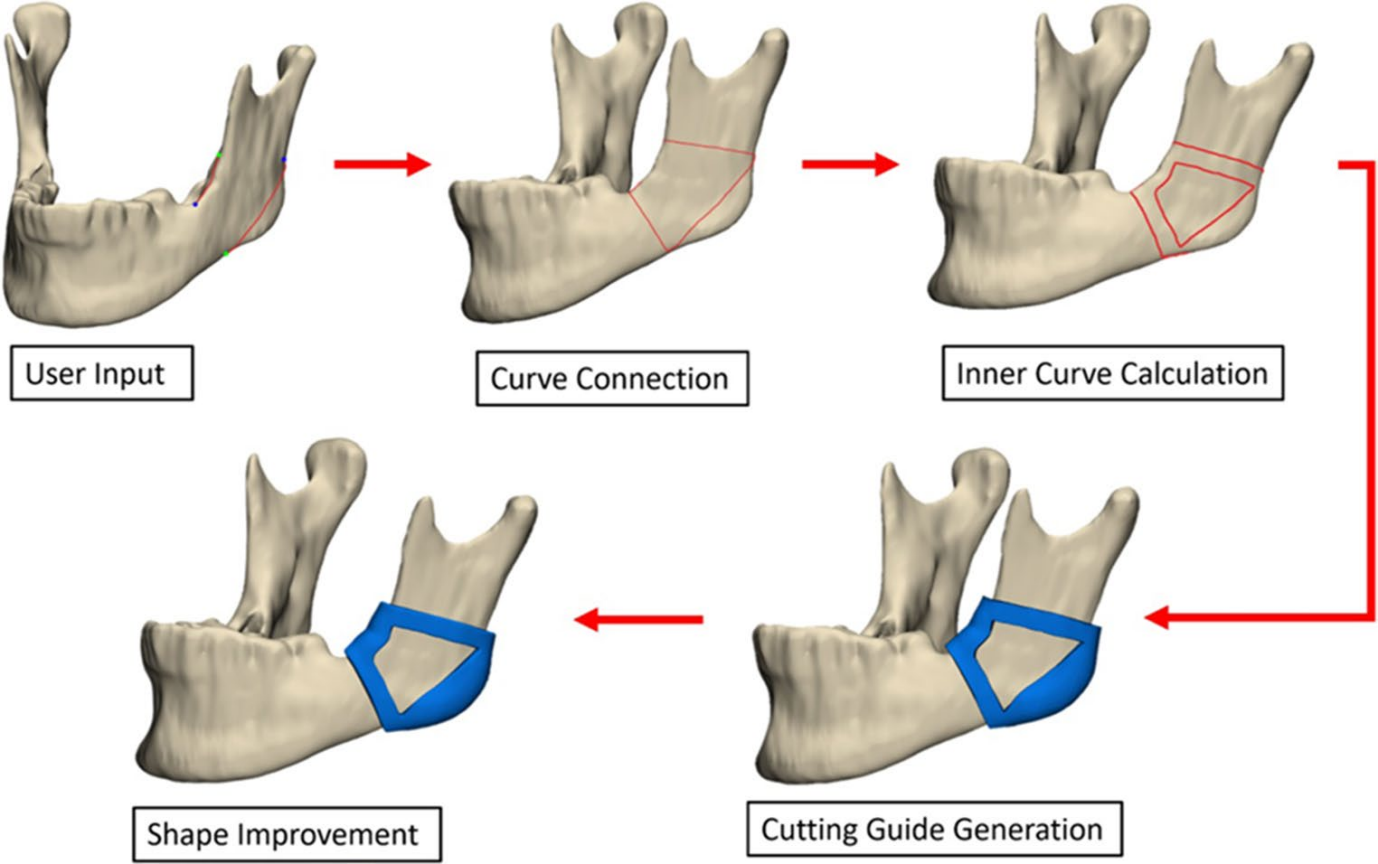
Representation of the workflow for the fully automated cutting guide generation in 3-Matic

The second model automatically connects the user-defined osteotomy and holding lines, smoothing and refining the curves to create a new inner curve. This process splits the mandibular surface into multiple new surfaces, from which the one representing the bottom of the cutting guide (CG) is identified, and an offset is applied. The resulting mesh was then exported as an STL file. The complete workflow is illustrated in Fig. 1.

The model allowed for easy design adjustments and greater reproducibility by modifying variable parameters, such as the guide’s thickness (3.0 mm), the holding arch’s width (6.0 mm), and the offset between the guide and mandible (0.35 mm).

### Manual workflow

**Fig. 2:**
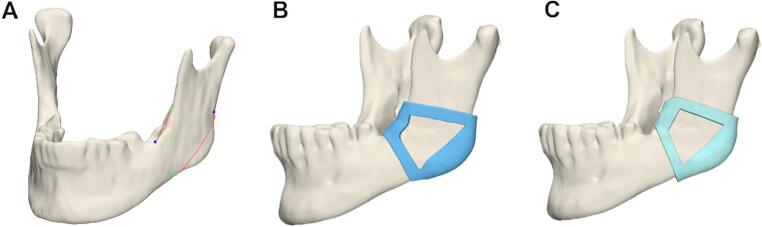
AI-segmented mandible (Case #27). (**A**) The required input curves (superior curve–holding line; inferior curve–osteotomy line) (**B**) the automatically designed guide (**C**) the manually designed guide

The selected 30 head and neck CT scans were imported into the Mimics Innovation Suite (Version 25.0, Materialise NV, Leuven, Belgium). Mandible segmentation was performed semi-automatically by two individuals: an expert researcher with extensive experience (having completed over 1,000 segmentations) and a beginner resident with limited experience (having completed 10 segmentations). The segmentation process utilized various tools, including Threshold, Split Mask, Region Grow, Edit Mask, Multiple Slice Edit, Smart Fill, and Smooth Mask. A clinician then performed virtual planning of the osteotomy line for each of the 60 mandibular angles. The holding line, which defines the position of the holding arch of the cutting guide, was placed near the linea obliqua, as shown in Fig. 2. The amount of mandible removed depended on the shape of the mandible. The osteotomy line for the cut was placed by the surgeon, following the experiences gathered in operating these cases. With this line, the guide could be planned. Next, the cutting guides were manually designed by both the expert (researcher, who had created over 100 cutting guides) and the novice (resident, with no prior experience in guide design). The guide was designed so that the cut performed would be at about 90 degrees to the surface of the mandible. The guides were created by performing a process known as “surface wrapping,” in which a 3D mesh of the mandible was wrapped with a digital surface model to enclose the mandible within a virtual shell. This wrapping process involved creating a boundary surface that conforms to the outer contour of the mandible. Once the wrapping was completed, the original mandible geometry was subtracted from this surface, leaving a hollow shell that represented the cutting guide. The final guide was obtained by trimming and refining this shell to ensure proper fit and functionality. All guides were subsequently exported as Standard Tessellation Language (STL) files.

### 3D printing

**Fig. 3:**
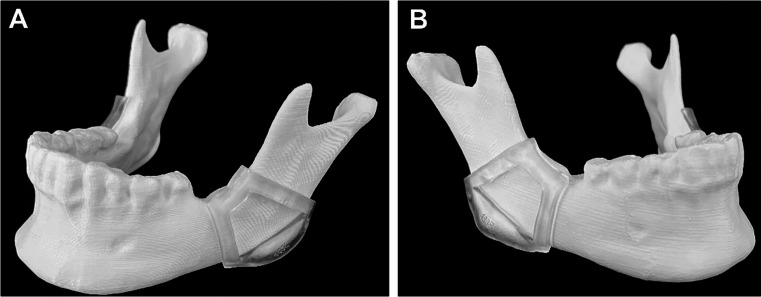
3D printed mandible with cutting guides. (**A**) Left and (**B**) right guide

The manually and automatically segmented mandibles were 3D-printed by fused filament fabrication with the MakerBot Replicator+ (MakerBot Industries, Brooklyn, USA) using polylactic acid (EcoPLA, 3DJAKE GmbH, Austria). The automatically and manually designed CGs were fabricated using a stereolithography 3D printer (Form 3B, Formlabs Inc., Somerville, MA, USA) and photopolymer resin material (Clear V4 Resin, Formlabs Inc., Somerville, MA, USA). Post-processing was done by immersing the guide in a 90% isopropyl alcohol solution in the Form Wash (Formlabs Inc., Somerville, MA, USA) for 20 min and UV curing with the Form Cure device (Formlabs Inc., Somerville, MA, USA) for 30 min. The supporting structures were removed with fine-cutting pliers. A printed mandible with its guides is displayed in Fig. 3.

### Comparisons

#### Segmentation comparison

**Fig. 4:**
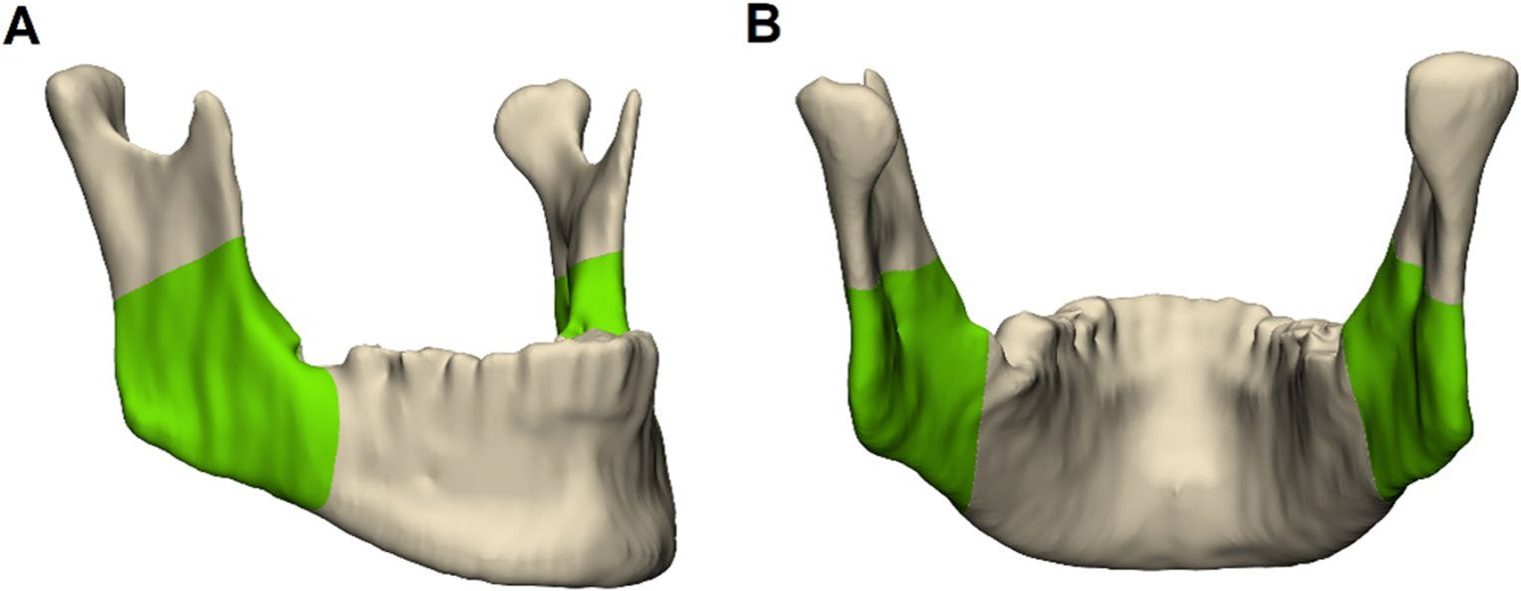
AI-segmented mandible (Case #27) with its ROI marked in green. (**A**) Fronto-lateral and (**B**) dorsal view

To assess the segmentation accuracy of the CNN, the AI-segmented mandibles were compared to the manually segmented ones in the ROI, as shown in Fig. 4. For each mandible, the right and left ROI were cut with manually placed planes at the top and bottom and individually assessed using a Python (Version 3.8.10) script. The dice similarity coefficient (DSC), mean surface distance (MSD), and Hausdorff distance (HD) were specified.

#### Cutting guide osteotomy line comparison

**Fig. 5:**
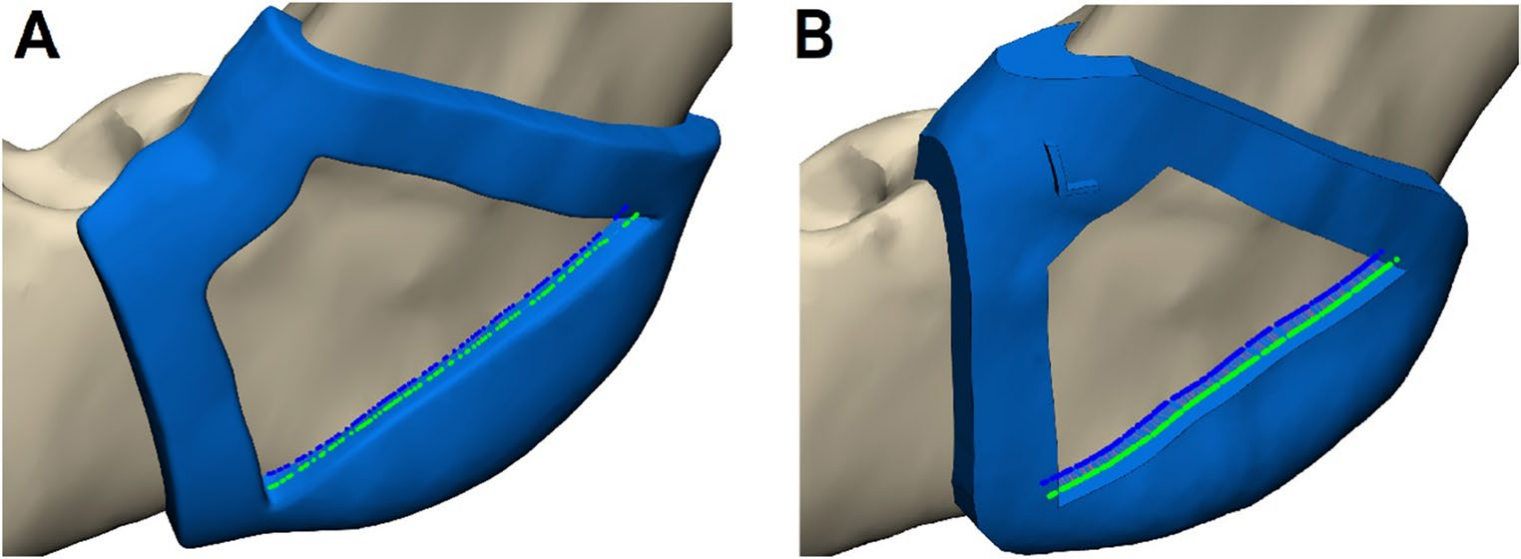
Cutting guides with perpendicular vectors to the mandibular surface. (**A**) Automatically generated guide and (**B**) manually designed guide

Two parallel lines were drawn on the perpendicular surface to the osteotomy line of each manually and automatically designed guide to determine the precision of the osteotomy line marked by the CG, as shown in Fig. 5. The vector from each point of the superior line to its closest point of the inferior line was calculated. The intersection points between each vector and the mandibular mesh generated a new line, which was compared to the osteotomy line by calculating the mean distance.

#### Cutting guide surface comparison

**Fig. 6:**
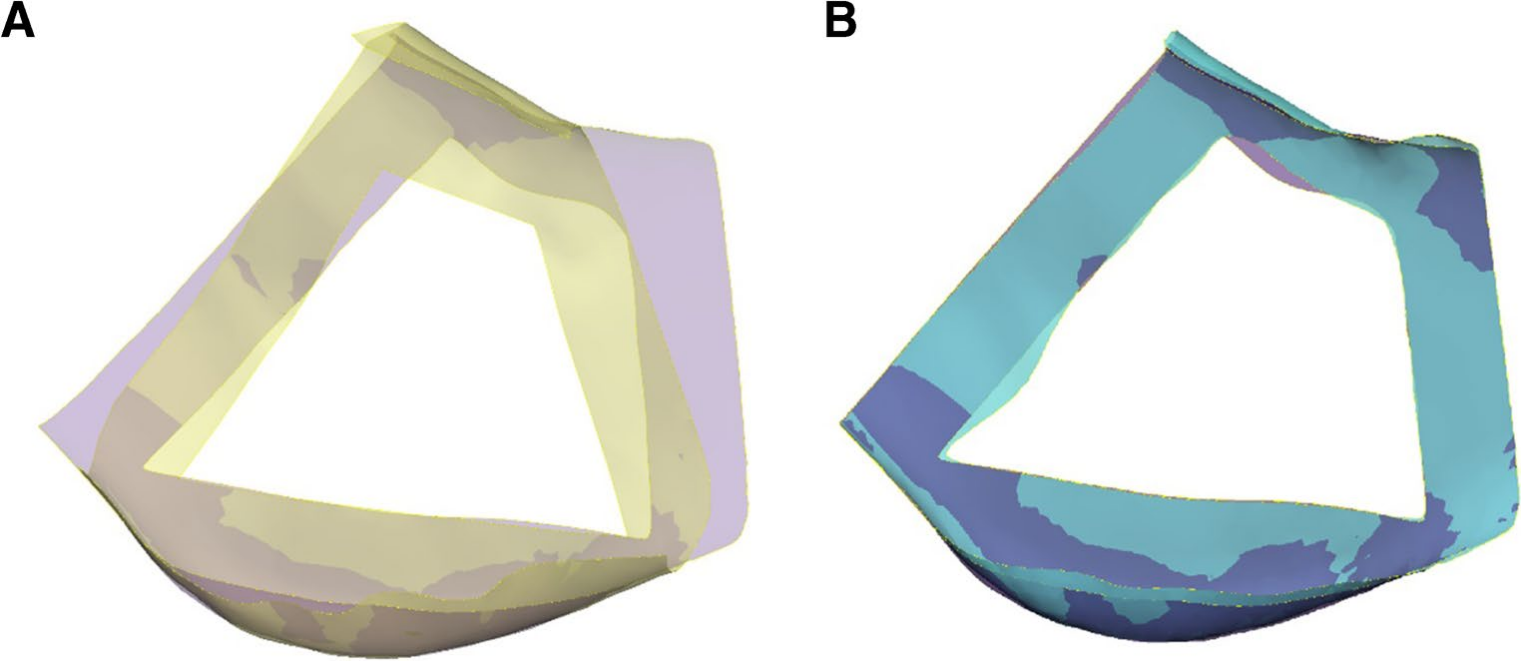
The inner surface of the cutting guide, (**A**) automatically generated guide on the manually segmented mandible (violet) in comparison to the manually generated guide (yellow) and (**B**) automatically generated guide on the manually segmented mandible (violet) in comparison to the automatically generated guide on the automatically segmented mandible (light blue)

The surfaces facing the mandible were matched with each other to compare the fit between the manually and automatically generated CGs. However, the automatically generated guides could not be directly compared to the manual guides due to variations in guide designs, resulting in differing surface shapes and dimensions. An intermediate step was implemented as a solution, developing automatically designed guides on the manually segmented mandible instead of the automatically segmented one. The evaluation process involved two steps. Firstly, the manually generated guides were compared to the automatically generated guides on the manually segmented mandible. Secondly, the automatically generated guides on the AI-segmented mandible were compared to the automatically segmented guides on the manual mandible, as displayed in Fig. 6. These assessments calculated the mean distance between the surfaces by determining the closest distance to the other surface for each point on a given surface.

### Workflow efficiency and timing

The segmentation and guide-generation success rates were reported to prove the workflow’s efficiency. The time for each process step was measured and compared to the manual workflow. Additionally, the elapsed time of the automated CG generation was compared to the design time of an expert and a novice user. The clinical usability of the automatically generated cutting guides was assessed by an experienced surgeon, who checked the fit and the design of the 3D-printed mandibles. In addition, a digital assessment of the accuracy of the osteotomy line defined by the CG about its intended position was performed.

### Statistical analysis

The data was collected and organized using Microsoft^®^ Excel^®^ from Microsoft 365 MSO (Microsoft Cooperation, Redmond, WA). Descriptive statistics such as normality testing, mean, standard deviation, median (Mdn), interquartile range (IQR), and a Mann-Whitney test were conducted using Prism (GraphPad Software, Inc.). Statistical significance was set at a p-value less than 0.05, denoted by an asterisk.

## Results

The results of the descriptive statistics analysis revealed that the data did not follow a normal distribution, as confirmed by the Shapiro-Wilk test (*p* < 0.05). As a result, the median was reported as a measure of central tendency instead of the mean.

### Segmentation accuracy

**Fig. 7:**
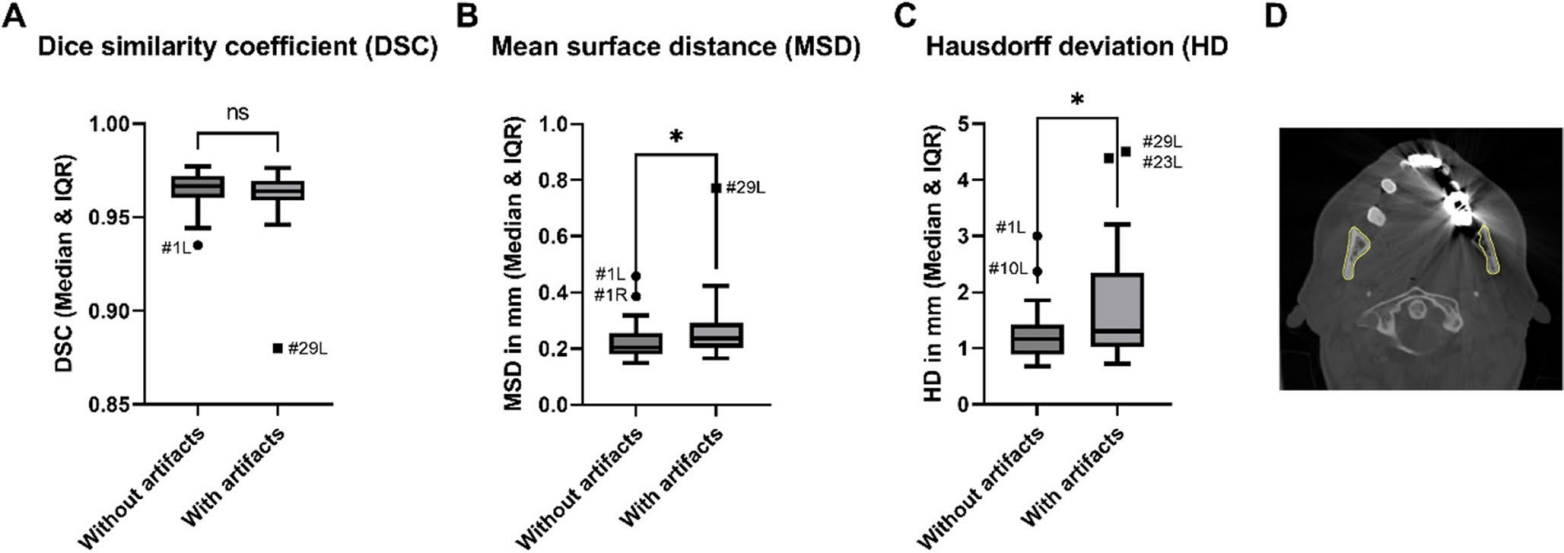
Segmentation accuracy measurements. (**A**) dice similarity coefficient (**B**) mean surface distance (**C**) Hausdorff deviation (**D**) axial slice of CT image with heavy artifacts and its segmentation (Case #29)

To evaluate the accuracy of the ROI segmentation, the manually segmented mandibular angles were assessed in comparison to those segmented by the 3D U-net. The segmentations show a high level of agreement, with a DSC ranging from 0.880 to 0.977 and a median value of 0.966. The MSD values ranged from 0.150 mm to 0.772 mm, with a median value of 0.212 mm, while the Hausdorff distance ranged from 0.677 mm to 4.51 mm, with a median value of 1.208 mm, as shown in Fig. 7. However, the left ROI of the CT image with the highest artifact level (Case #29) consistently exhibited deviant outcomes in all analyses conducted, as did the left ROI of Case #1. Visual inspection revealed that the segmentation procedure failed to delineate the relevant anatomical structures accurately in both cases—the extended list of the measured metrics is shown in the Table of Online Resource 1.

### Workflow success rate

The success rate of the segmentation and the CG generation was evaluated to assess the robustness of the fully automated, AI-based, digital workflow. A total of 30 segmentations were performed, of which all were successful and only two requiring manual adjustments (Cases #1 and #29). Subsequently, we generated cutting guides for each ROI with our automated system, demonstrating an error-free performance in all cases.

### Fit of cutting guide

#### Osteotomy line accuracy

**Fig. 8:**
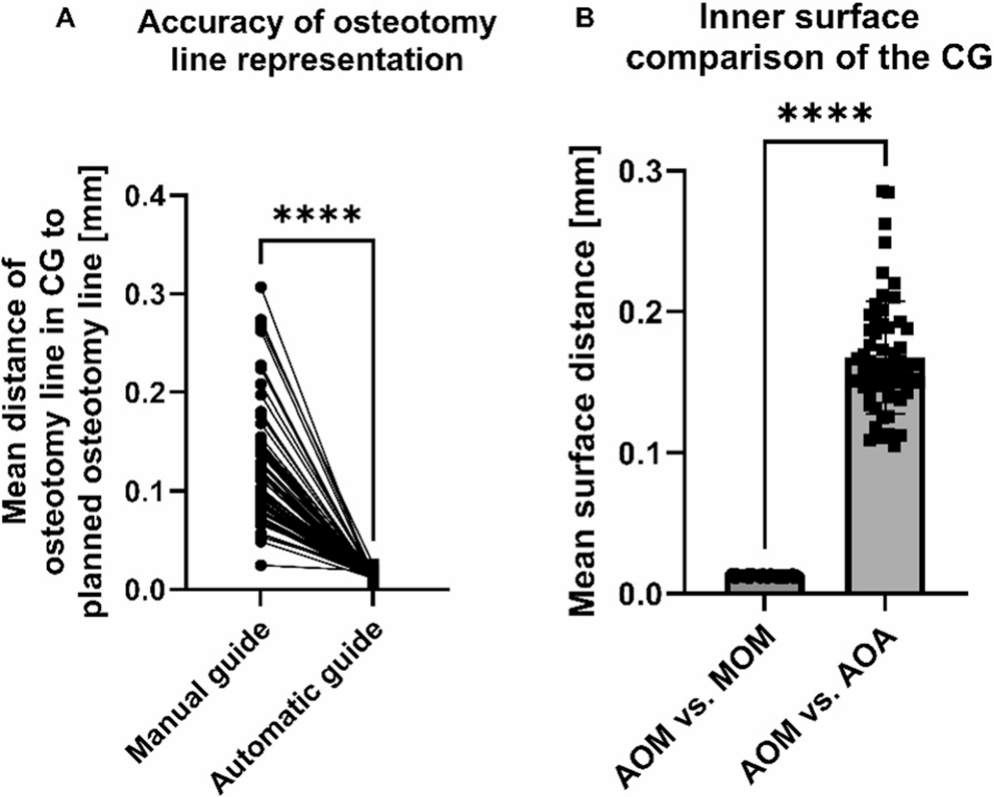
(**A**) Accuracy of the osteotomy line representation in manually and automatically designed guides. (**B**) Comparison of the inner mandible-facing surface of the cutting guides (automatically generated guides on the manually segmented mandible (AOM), automatically generated guides on the automatically segmented mandible (AOA), and manually designed guides on the manually segmented mandible (MOM))

We assessed the mean distances between the osteotomy line, indicated by the cutting guides, and the planned osteotomy curve to evaluate the accuracy of the guides. The comparison shows that the median distance between the curves for the automatic guide was 0.012 mm (IQR: 0.010 mm to 0.015 mm), whereas for the manually designed guides, the median distance was 0.112 mm (IQR: 0.084 mm to 0.147 mm), as shown in Fig. 8.

#### Surface comparison

The surface comparison between the automatically generated guides on the AI-segmented mandible and the automatically generated guides on the manually segmented mandible resulted in a median mean distance of 0.156 mm (IQR: 0.144 mm to 0.188 mm), whereas the surface comparison between the manually designed guides and the automatically designed guides on the manually segmented mandible resulted in a median mean distance of 0.013 mm (IQR: 0.012 mm to 0.013 mm), as shown in Fig. 8.

#### Fit of the cutting guide

The experienced surgeon found that all 3D-printed, automatically generated cutting guides fit onto their respective mandibles with good retention. Further, all the guides were found to be clinically applicable.

### Timing

**Fig. 9:**
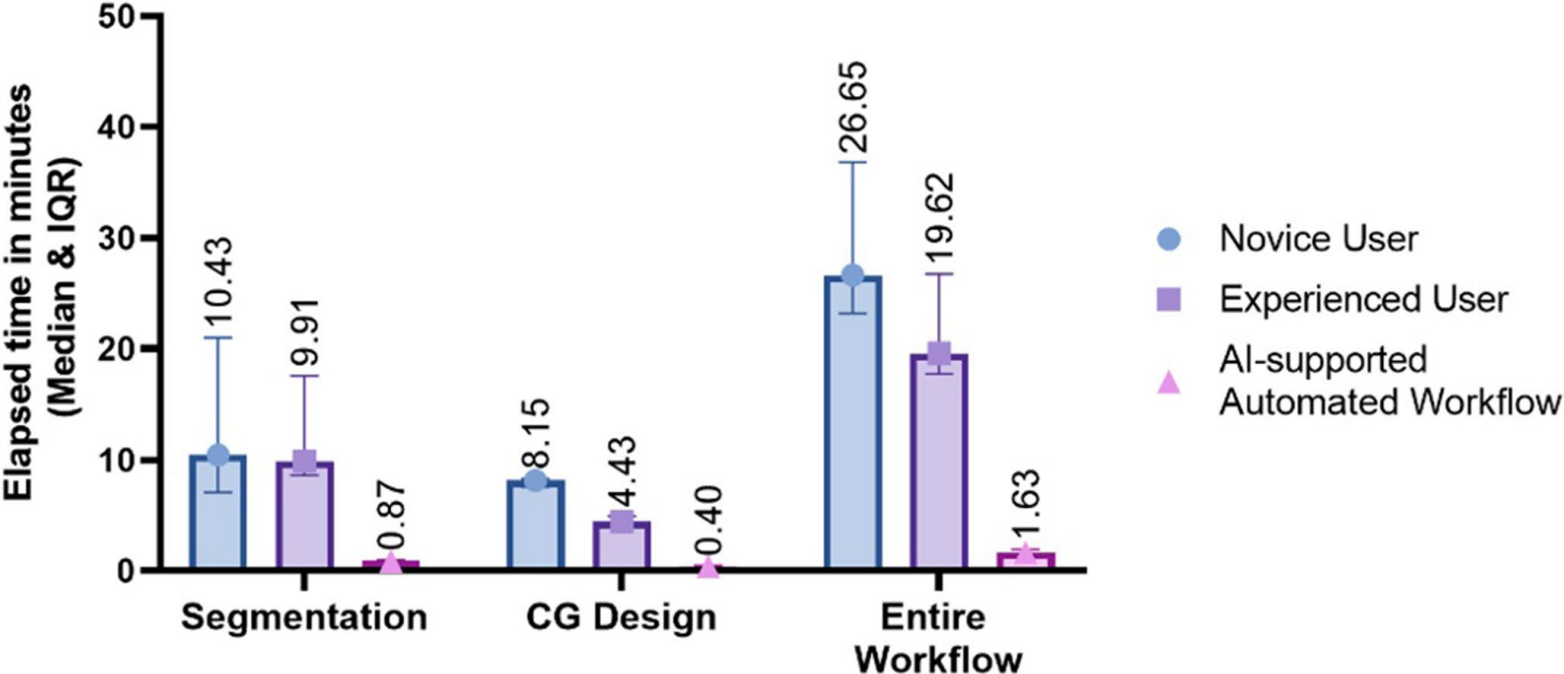
Elapsed time of segmentation, cutting guide design, and entire workflow performed by novice, experienced user vs. automated workflow

The workflow durations for 30 cases and 60 cutting guides were recorded, comparing manual and AI-supported methods. For segmentation, manual efforts required a median of 9 min 54 s for the expert and 10 min 26 s for the novice, while AI segmentation, including necessary retouching, took only 1 min 2 s. Cutting guide design followed a similar trend, with manual designs taking 4 min 26 s for the expert and 8 min 9 s for the novice, compared to just 24 s for the automated system. For the complete workflow, including segmentation and cutting guide generation, the expert needed 19 min 37 s, and the novice required 26 min 39 s. In contrast, the AI-supported workflow was completed in 1 min 38 s. This resulted in time savings of 17 min 27 s for the expert and 24 min 44 s for the novice. Overall, the automated system was 10.2 times faster than the expert and 16.3 times more efficient than the novice. The timings are displayed in Fig. 9.

## Discussion

This study presents an AI-supported digital workflow for the design of patient-specific cutting guides for mandible osteotomies in facial feminization surgery (FFS).

At present, there are no reported fully automated systems for virtual surgical planning in facial feminization surgery (FFS). Planning remains largely manual, requiring individualized anatomical assessment and surgeon-driven simulation. In comparison, semi-automated workflows have been developed for orthognathic surgery and cranio-maxillofacial (CMF) reconstructions, such as mandibular and midfacial defect rehabilitation. Commercial platforms like IPS^®^ CaseDesigner^®^ (KLS Martin Group) and Mimics Enlight CMF (Materialise NV) provide structured digital environments supporting segmentation, osteotomy simulation, and splint design. However, despite improved workflow standardization, substantial user input is still required for landmark placement, segmentation, and movement simulation, and true automation is not yet realized.

Recent work, such as the cloud-based digital workflow proposed by Lee et al.​ [31], demonstrates progress towards partial automation, particularly in data management and semi-automated registration. Nevertheless, critical steps remain manual, and fully AI-driven solutions for segmentation, movement planning, and soft tissue prediction are lacking. Thus, while current systems enhance reproducibility and efficiency, significant opportunities exist to develop fully automated, intelligent planning frameworks for broader CMF surgical applications.

The workflow developed in this study incorporates two key innovations: automated segmentation of the mandible and automated cutting guide generation, each contributing independently to the efficiency and accuracy of the process.

The AI-segmentation model achieved a high overall DSC value of 0.966 in the region of interest (ROI), indicating good performance in predicting segmentation that closely matches the ground truth and is comparable to the deviations among inter-raters [32]. However, the higher HD values indicate areas where the predicted segmentation differs from the ground truth. It is worth noting that the nerve channel, which can be segmented or filled in different ways, is located within the selected ROI. Thus, higher values for the HD were expected. Nevertheless, these differences were acceptable due to the position of the buccally placed guides.

Regarding the cutting guide generation, the automatically designed guides demonstrated significantly higher accuracy in representing the osteotomy line. The median distance between the planned osteotomy and the osteotomy line of the automated guides was just 0.012 mm, compared to 0.112 mm for the manually designed guides. This precision was achieved due to the mathematically determined offset, resulting in a 90-degree angle between the side surface of the cutting guide and the mandibular surface. The inner surface comparison revealed that, when generating the CGs on the same mandible, the mean distance between the inner surface of the manually and automatically designed guide only differs by 0.013 mm (AOM vs. MOM comparison). Consequently, the primary source of any deviation in the inner surface of the cutting guides can be attributed to the underlying mandibular shape on which they are designed. The mean surface distance between automatically created guides on the manual and AI-segmented mandible was calculated to be 0.156 mm (AOM vs. AOA comparison). This value is comparable to the median of the mean surface distance in the AI-based mandible segmentation within the region of interest (ROI), which amounted to 0.212 mm.

This study showed the benefit of using AI and the potential to improve efficiency and reduce the time for mandible osteotomy procedures. The automated workflow generated cutting guides in approximately 1 min and 30 s per case (mandible segmentation and guide design for both angles). The automated workflow outperformed the manual approach, even in the two cases where minor segmentation errors had to be corrected afterward. Compared to a fully manual workflow, the AI-supported workflow is 16.3 times faster than a novice user and 10.2 times faster than an experienced user. This improvement results in a median time saving of 25 min, or 17 min per case, for the novice and expert user, respectively. The production of a cutting guide design depends on extensive knowledge of CAD/CAM software and virtual surgical planning, which in turn relies on the user’s ability to quickly and properly acquire this expertise [33]. Our automated workflow only requires two curves on the mandibular angle as input, enabling inexperienced users and clinicians to plan surgeries independently of an external service provider and avoiding waiting times, miscommunication, and delays. The higher accessibility and time savings of automated workflows are clinically applicable and relevant, particularly considering the growing need for efficient and cost-effective healthcare services.

Our effort to understand, master, and implement AI technologies in CMF workflows also comes in accordance with the Dublin Declaration on Human Resources for Health (2017), which stated a shortage of around 40 million healthcare workers by 2030. AI-driven technologies are already optimizing costs and reducing biases, as shown in other sectors, e.g., the financial and automotive sectors [34, 35]. However, further studies are needed for evidence-based medical practice.

The fact that the ground truth was based on only one expert’s manual segmentation may be a limitation of the study. Therefore, we recognize that further research is needed to establish the reliability of this approach.

Future research should optimize the 3D U-net for segmentations to improve the workflow’s accuracy, efficiency, and reliability in a clinical setting. Automatic checking for nerve interference could also be implemented. Alternatively, commercially available AI-supported segmentation software could be used.

Further validation through clinical trials is necessary to confirm the accuracy and effectiveness of the cutting guides in vivo and assess the clinical outcomes and impact on patient satisfaction and healthcare costs.

## Conclusion and outlook

To our knowledge, this paper proposes the first fully automated digital workflow to fabricate cutting guides for mandible osteotomies for facial feminization surgery using CAD/CAM software and 3D printing at the point of care. We developed an AI-based segmentation software using a convolutional neural network and integrated it into a digitalized workflow to automatically create cutting guides with minimal manual input. The study demonstrates the feasibility and efficiency of the proposed approach by generating accurate cutting guides, which can help improve patient outcomes. The paper contributes to the ongoing efforts to enhance gender-affirming care for transgender patients through innovative technological solutions.

Artificial intelligence has the potential to optimize cutting guide generation for FFS, streamline the designing process, and be implemented in fully automated workflows without manual input. It is essential to continue exploring and refining these technologies to improve patient outcomes, enhance surgical precision, and increase efficiency in healthcare delivery.

## Electronic supplementary material

Below is the link to the electronic supplementary material.

Supplementary Material 1 (DOCX 20.1 KB)

## Data Availability

The datasets used and/or analyzed during the current study are available from the corresponding author on reasonable request.

## Abbreviations

3D: Three-dimensional
AI: Artificial intelligence
CG: Cutting guide
CNN: Convolutional neural network
CT: Computed tomography
DICOM: Digital imaging and communications in medicine
DSC: Dice similarity coefficient
IAN: Inferior alveolar nerve
IQR: Interquartile range
MSD: Mean surface distance
HD: Hausdorff distance
ROI: Region of interest
STL: Standard tessellation language
