# Supplementary material for: Innovations in gender affirmation: AI-enhanced surgical guides for mandibular facial feminization surgery
Source: Clin Oral Investig. 2025 Jul 25;29(8):390. doi: 10.1007/s00784-025-06459-2 (PMC12296787; doi:10.1007/s00784-025-06459-2)
Supplement: Supplementary file 1 — (DOCX 20.1 KB) [file 784_2025_6459_MOESM1_ESM.docx]

**Supplementary Information (SI)**

| Assigned name | DSC | MSD (mm) | HD (mm) |
| --- | --- | --- | --- |
| #16L | 0.9766 | 0.1798 | 2.0000 |
| #16R | 0.9772 | 0.1685 | 1.4142 |
| #17L | 0.9602 | 0.2615 | 2.4495 |
| #17R | 0.9642 | 0.2746 | 2.4495 |
| #18L | 0.9738 | 0.1686 | 2.0000 |
| #18R | 0.9661 | 0.2195 | 1.7321 |
| #19L | 0.9627 | 0.2228 | 2.2361 |
| #19R | 0.9530 | 0.2713 | 2.4495 |
| #20L | 0.9719 | 0.1866 | 3.0000 |
| #20R | 0.9679 | 0.2042 | 2.0000 |
| #21L | 0.9608 | 0.2403 | 4.0000 |
| #21R | 0.9633 | 0.2223 | 2.4495 |
| #22L | 0.9620 | 0.2334 | 3.6056 |
| #22R | 0.9582 | 0.2765 | 2.4495 |
| #23L | 0.9459 | 0.4233 | 8.0000 |
| #23R | 0.9625 | 0.2686 | 2.8284 |
| #24L | 0.9654 | 0.2130 | 3.0000 |
| #24R | 0.9647 | 0.2682 | 6.7082 |
| #25L | 0.9684 | 0.2025 | 2.2361 |
| #25R | 0.9514 | 0.3512 | 8.6023 |
| #26L | 0.9749 | 0.1652 | 3.1623 |
| #26R | 0.9741 | 0.1729 | 2.2361 |
| #27L | 0.9665 | 0.1854 | 2.2361 |
| #27R | 0.9483 | 0.3183 | 7.5498 |
| #28L | 0.9763 | 0.2129 | 2.2361 |
| #28R | 0.9715 | 0.2477 | 3.0000 |
| #29L | 0.8799 | 0.7722 | 8.0623 |
| #29R | 0.9620 | 0.2964 | 7.6811 |
| #30L | 0.9679 | 0.2043 | 2.2361 |
| #30R | 0.9699 | 0.1999 | 1.4142 |

**Table, Online Resource 1.** AI-Segmentation accuracy measurements (dice similarity coefficient (DSC), mean surface distance (MSD) and Hausdorff distance (HD)).

| Assigned name | DSC | MSD (mm) | HD (mm) |
| --- | --- | --- | --- |
| #1L | 0.9349 | 0.4570 | 6.4031 |
| #1R | 0.9442 | 0.3862 | 3.3166 |
| #2L | 0.9732 | 0.1820 | 2.4495 |
| #2R | 0.9762 | 0.1616 | 1.7321 |
| #3L | 0.9633 | 0.2653 | 7.0000 |
| #3R | 0.9643 | 0.2601 | 3.0000 |
| #4L | 0.9767 | 0.1728 | 5.9161 |
| #4R | 0.9743 | 0.1900 | 2.4495 |
| #5L | 0.9684 | 0.2131 | 2.2361 |
| #5R | 0.9679 | 0.2105 | 2.2361 |
| #6L | 0.9710 | 0.1616 | 4.0000 |
| #6R | 0.9644 | 0.1909 | 4.1231 |
| #7L | 0.9693 | 0.1741 | 2.2361 |
| #7R | 0.9481 | 0.3170 | 2.0000 |
| #8L | 0.9628 | 0.2088 | 3.1623 |
| #8R | 0.9480 | 0.3177 | 9.1104 |
| #9L | 0.9750 | 0.1498 | 1.4142 |
| #9R | 0.9680 | 0.1918 | 2.0000 |
| #10L | 0.9533 | 0.2257 | 6.4807 |
| #10R | 0.9534 | 0.2052 | 4.1231 |
| #11L | 0.9710 | 0.1797 | 2.2361 |
| #11R | 0.9721 | 0.1778 | 1.4142 |
| #12L | 0.9608 | 0.2362 | 3.0000 |
| #12R | 0.9643 | 0.2082 | 2.0000 |
| #13L | 0.9721 | 0.1629 | 5.0990 |
| #13R | 0.9671 | 0.1919 | 2.2361 |
| #14L | 0.9655 | 0.2027 | 2.4495 |
| #14R | 0.9673 | 0.1976 | 2.2361 |
| #15L | 0.9595 | 0.2742 | 5.7446 |
| #15R | 0.9580 | 0.2449 | 4.0000 |
